# The Condition of the Pupil in Acute Alcoholic Poisoning

**Published:** 1903-12-19

**Authors:** 


### THE CONDITION OF THE PUPIL IN ACUTE ALCOHOLIC POISONING.

This is a question of some practical importance, inasmuch as the state of the pupils is one of the aids to diagnosis in all conditions of unconsciousness of more or less sudden development. The problem offered by a person found in a state of coma is always a very pressing one, and its solution is not always an easy task. Appeal is invariably made, *inter alia*, to the condition of the pupils, and in reference to the significance of the facts there observed there is for the most part general agreement. But it is not so in the case of poisoning by alcohol. Everyone recognises the pin-point pupil of opium, the contracted pupils of hæmorrhage into the pons Varolii, the contraction which attends carbolic acid poisoning, the suggestion of an organic lesion which arises from inequality of the pupils, and so on. But authoritative statements dealing with the condition of the pupils in alcoholic poisoning show considerable lack of decision and are in many instances even mutually contradictory. In some manuals the pupils are said to be contracted, in others they are described as dilated, whilst in one well-known text-book the statement runs that they are "either contracted or dilated—frequently contracted at first and afterwards dilated." This is certainly very confusing, and it introduces an element of ambiguity into a situation in which decision carries very anxious responsibilities. The present uncertainty is the more surprising seeing the question was carefully investigated so long as twenty-five years ago, and that, too, with a very precise result. We refer to the observations of Dr. (now Sir William) Macewen, published in the year 1879. Macewen found, as the result of a long series of cases, that in alcoholic coma the pupil is contracted, provided that the patient has not been recently disturbed. When, however, any external stimulus is applied, as for example, in efforts made to rouse the patient by slapping, moving or shaking him, pulling his hair or ears, etc., the pupils dilate. If now the patient is allowed to be quiet, the pupils after an interval varying in different cases from 5 to 20 minutes gradually again become contracted. These observations are of great value, but they do not seem to have attracted their proper share of attention. We note, however, that in his recently-published text-book, Professor Glaister, after a police practice of some eighteen years, describes Macewen's statements as "absolutely reliable in cases of pure alcoholic coma."

